# Pancreatic Iodine Density and Fat Fraction on Dual-Energy Computed Tomography in Acute Pancreatitis

**DOI:** 10.3390/diagnostics14090955

**Published:** 2024-05-02

**Authors:** Zrinka Matana Kaštelan, Ivan Brumini, Goran Poropat, Lovro Tkalčić, Tiana Grubešić, Damir Miletić

**Affiliations:** 1 Department of Diagnostic and Interventional Radiology, Clinical Hospital Center Rijeka, Kresimirova 42, 51000 Rijeka, Croatiadamir.miletic@medri.uniri.hr (D.M.); 2 Department of Anatomy, Faculty of Medicine of the University of Rijeka, Brace Branchetta 20, 51000 Rijeka, Croatia; 3 Department of Radiological Technology, Faculty of Health Studies of the University of Rijeka, Ul. Viktora Cara Emina 5, 51000 Rijeka, Croatia; 4 Department of Gastroenterology, Clinical Hospital Center Rijeka, Kresimirova 42, 51000 Rijeka, Croatia; 5 Department of Internal Medicine, Faculty of Medicine of the University of Rijeka, Brace Branchetta 20, 51000 Rijeka, Croatia; 6 Department of Radiology, Faculty of Medicine of the University of Rijeka, Brace Branchetta 20, 51000 Rijeka, Croatia

**Keywords:** computed tomography, fat, iodine, pancreas, pancreatitis

## Abstract

The aim of our study was to investigate iodine density (ID) and fat fraction (FF) on dual-energy computed tomography (DECT) in patients with acute pancreatitis (AP). This retrospective study included 72 patients with clinically confirmed AP and 62 control subjects with DECT of the abdomen. Two radiologists assessed necrosis and measured attenuation values, ID, and FF in three pancreatic segments. We used receiver operating characteristic (ROC) analysis to determine the optimal threshold for ID for the differentiation between AP groups. The ID was significantly higher in interstitial edematous AP compared to necrotizing AP and the control group (both *p* < 0.05). The ROC curve analysis revealed the thresholds of ID for detecting pancreatic necrosis ≤ 2.2, ≤2.3, and ≤2.4 mg/mL (AUC between 0.880 and 0.893, *p* > 0.05) for the head, body, and tail, respectively. The FF was significantly higher for pancreatitis groups when compared with the control group in the head and body segments (both *p* < 0.001). In the tail, the difference was significant in necrotizing AP (*p* = 0.028). The ID values were independent of attenuation values correlated with the FF values in pancreatic tissue. Iodine density values allow for differentiation between morphologic types of AP.

## 1. Introduction

Acute pancreatitis (AP) is a relatively common acute inflammatory disease of the pancreas, ranging from mild and self-limiting to a severe form that is associated with local and systemic complications and can lead to death [1]. The diagnosis is established when two of the following three criteria are fulfilled: (1) typical acute abdominal pain in the epigastric region, often radiating to the back, (2) an increase in serum amylase or lipase, exceeding three times the upper limit of normal, and (3) characteristic imaging findings using ultrasound (US), computed tomography (CT), or magnetic resonance imaging (MRI) [2]. The revised Atlanta classification from 2012 differentiates two morphological types of AP, namely, interstitial edematous and necrotizing pancreatitis, and three categories of severity: mild, moderately severe, and severe AP [3]. Regarding the onset of clinical symptoms, AP is divided into an early phase (limited to the first seven days) and a late phase (which may last weeks or even months), although these phases may overlap [3,4]. Various clinical scoring systems, such as Ranson, Acute Physiology and Chronic Health Evaluation II (APACHE II), and Bedside Index for Severity in AP (BISAP) [5,6,7], and radiological scoring systems, such as CT severity index (CTSI) and modified CT severity index (mCTSI) [8,9], are used for the classification and the severity stratification of AP. Clinical and laboratory data are bases for calculating clinical scoring systems, and in radiological scoring systems, the focus is on imaging features of AP.

Radiological severity often corresponds to clinical severity. However, some patients with interstitial edematous pancreatitis may also develop severe AP, and patients with necrotizing pancreatitis may present with an uncomplicated clinical course [2].

The timely and adequate treatment of patients with AP impacts the outcome, especially in patients with severe AP [10,11]. Contrast-enhanced CT (CECT) remains the preferred imaging modality and plays an important role in deciding on the treatment of morphologically severe AP [12,13]. The optimal window for evaluating pancreatic tissue damage and the development of potential local complications caused by AP using conventional single-energy CT (SECT) is between the third and the seventh day of the first week following symptom onset [4,13,14]. On SECT, which relies on attenuation differences in different tissues, morphological changes in affected parenchyma may be subtle and demanding to distinguish from unaffected parts, especially in the mild form and in the early phase, leading to an underestimation of the extent of the disease [9]. In contrast, dual-source dual-energy CT (DECT) uses two X-ray beams at different tube energies, followed by complex computer algorithms which, along with blended greyscale images that correspond to SECT images for routine clinical interpretation, allow for material decomposition and characterization [15,16]. The result are more quantitative data than are obtained by means of SECT [17]. Dual-energy CT with different post-processing options enables a more accurate assessment of various pancreatic pathologies and can used for the detection and evaluation of focal pancreatic lesions, solid and cystic, for the distinction of adenocarcinoma from chronic mass-forming pancreatitis, and in the assessment of AP [18]. With DECT using iodine quantification, the assessment of AP has higher sensitivity when compared to standard image evaluation for establishing diagnosis and severity stratification [19,20].

This study aimed to investigate the potential additional value of DECT to better understand pancreatic tissue damage in interstitial edematous and necrotizing types of AP using differences in iodine density (ID) and fat fraction (FF) in patients with AP and control subjects without pancreatic diseases. As far as we know, this is the first study that analyzed ID and FF in different pancreatic segments (pancreatic head, body, and tail) separately in patients with AP.

## 2. Materials and Methods

### 2.1. Study Population

After obtaining approval from the institutional review board with a waiver of informed consent, a single-center retrospective study was performed.

We searched our hospital database for consecutive patients with a clinical diagnosis of AP who were referred to DECT between November 2019 and May 2022 by in-hospital gastroenterologists. Included patients were older than 18 years with the first episode of AP in a lifetime. In-hospital gastroenterologists made the diagnosis according to the criteria of the revised Atlanta classification from 2012 [3]. For the study, the gastroenterologist prospectively assigned the APACHE II scores [6]. The first DECT exam during the same hospital stay in the same patient was analyzed. The exclusion criteria were follow-up examinations in the same patients during the same hospital stay (n = 10), recurrent AP or AP in patients with chronic pancreatitis (n = 20), known or newly diagnosed underlying pancreatic tumor, and previous pancreatic surgery (n = 4), and patients with symptoms consistent with AP lasting longer than one month (n = 1). The final study group of patients with AP consisted of 72 patients (mean age 65 ± 16 years; range 22–91 years).

**Figure 1:**
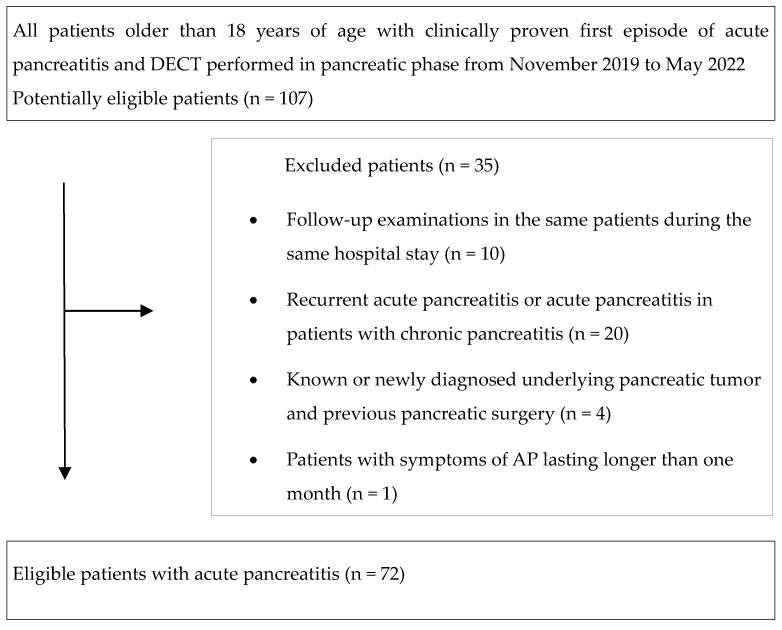
Standards for Reporting of Diagnostic Accuracy (STARD 2015) flowchart of included patients with acute pancreatitis.

The flowchart of patients with AP included in the study was based on the Standards for Reporting of Diagnostic Accuracy (STARD 2015) guidelines (Figure 1).

The characteristics of patients with AP, including demographic data (age and gender), body mass index (BMI), clinical score (APACHE II), the length of hospital stay, and in-hospital mortality, were retrieved from electronic medical records.

The control group included 62 consecutive patients without (known or newly detected) pancreatic disease, scanned with a DECT protocol that included a pancreatic phase in dual-energy mode. The indication for CT in these patients was the further evaluation of incidentally detected focal hepatic lesions by the US and staging of malignant tumors (colorectal carcinoma, hepatocellular carcinoma, cholangiocarcinoma, and lung carcinoma).

### 2.2. CT Protocol and Image Reconstruction

All DECT examinations were performed on the third-generation dual-source scanner (SOMATOM Flash, Siemens Healthineers, Forchheim, Germany). The scanner automatically creates blended greyscale images. The Liver VNC (syngo.CT Dual Energy, Siemens Healthineers, Forchheim, Germany), based on a 3-material decomposition algorithm, generates material decomposition images.

Participants were scanned in a supine position, with anteroposterior and lateral scout, craniocaudally from the diaphragm to the iliac crest in inspiratory breath-hold, 40 s after an injection (pancreatic phase) of non-ionic iodinated contrast agent through the cubital vein, at a flow rate of 3.5 mL/s, with a dose of 1.2 mL/kg up to a max. of 120 mL, followed by 30 mL of saline. Tube 1 was set at 100 kV, and tube 2 at 140 kV, with rotation time 0.5 s, pitch 0.9, and collimation 2 × 128 × 0.6 mm. Participants were not given oral contrast media. Automatic attenuation-based tube current modulation (CARE Dose 4D) was set on. An iterative reconstruction algorithm (Sapphire 3, Siemens Healthineers, Forchheim, Germany) was used.

### 2.3. Image Analysis

Two radiologists with 12 and 5 years of experience in abdominal radiology (5 years of experience in DECT for both) independently assessed DECT examinations of patients with AP and control subjects. The readers were aware of the existence of AP, or lack thereof, but were blinded to routine radiology reports, clinical data, APACHE II scores, and biochemical results.

The images were analyzed at a dedicated 3D multi-modality workstation (syngo.via, version VB60A, Siemens Healthineers) in the axial plane with a 3 mm slice thickness.

For the calculation of mCTSI [9], blended greyscale images with a weighted ratio of 50% to 50% of the 100 kV and 140 kV dual-energy data were used. In a blended image, the low and high kVp datasets are combined to simulate a conventional image. This image is considered equivalent to SECT images [18]. The differences in measurements of mCTSI exceeding 5% were resolved by consensus.

**Figure 2:**
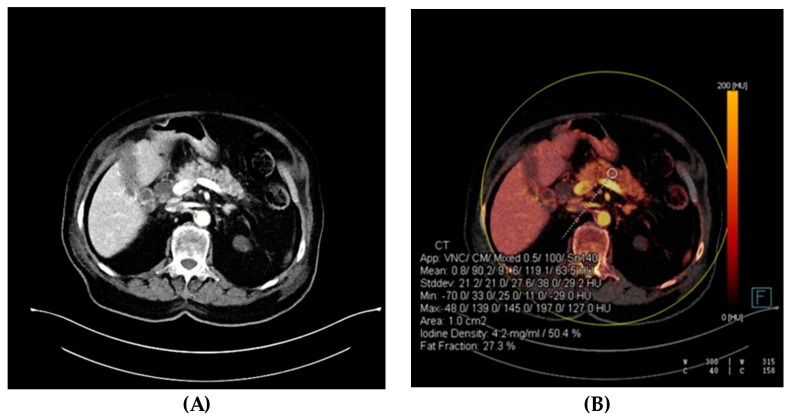
Dual-energy computed tomography (DECT) quantification. Axial images of a 91-year-old female patient with acute interstitial edematous pancreatitis. APACHE II = 13, mCTSI = 2. (**A**) Blended greyscale image showed mild gland enlargement. (**B**) Iodine overlay image with region-of-interest (ROI) and quantification of attenuation values, iodine density, and fat fraction. APACHE II, Acute Physiology and Chronic Health Evaluation II; mCTSI, modified CT severity index.

For the qualitative assessment of the viability of pancreatic tissue and region-of-interest (ROI) measurements, post-processed iodine overlay images generated by the Liver VNC algorithm (Siemens Healthineers, Forchheim, Germany) were used. In iodine overlay images, the iodine content is color-coded with a gradient and superimposed on greyscale virtual-non-contrast (VNC) images, and absent enhancement indicates pancreatic necrosis [21]. Additionally, this post-processing algorithm in selected areas (ROIs) provides calculated data for the quantification of attenuation values (VNC and mixed Hounsfield units [HU]), ID, and FF [22]. Two readers manually placed circular ROIs of 1 cm^2^ in three different segments of the pancreas (head, body, and tail) in each participant, avoiding vessels, parenchymal calcifications, and surrounding structures (Figure 2). In patients with qualitatively assessed necrosis, the readers placed ROIs within.

### 2.4. Statistical Analysis

A statistical analysis of data was performed using Statistica for Windows, release 14.0.1.25 (Statsoft, Inc., Tulsa, OK, USA). The normality of the distribution of ID, FF, mixed HU, VNC, BMI, APACHE II, and mCTSI was verified using the Kolmogorov–Smirnov test. Although some of these variables were not normally distributed (BMI, APACHE II, mCTSI, and ID measured in the head), we used mean ± standard deviation (SD) to present all data because it allows easier understanding and comparison with other studies. Two groups of patients were compared using the Mann–Whitney test or t-test for independent samples, respectively, and one-way ANOVA or the Kruskal–Wallis test was used to evaluate the differences between the three patient groups according to the investigated variables. Post hoc analysis was performed using either Tukey’s test or the Mann–Whitney test. The association between variables was calculated by means of Spearman rank or Pearson correlation coefficient. Inter-rater reliability was established by the intraclass correlation coefficient (ICC) grading system developed by Koo TK and Li MY (poor: <0.40; fair: 0.40–0.59; good: 0.60–0.74; excellent correlation: 0.75–1.00) [23]. The correct ICC form for inter-rater reliability included a two-way random-effects model and absolute agreement. Receiver operating characteristic (ROC) analysis was used to calculate the ROC plot and the overall sensitivity and specificity of ID. The area under the ROC curve (AUC) was a measure of how well the ID discriminates between interstitial edematous and necrotizing AP groups. All statistical values were considered significant at a *p*-value (*p*) < 0.05 level.

## 3. Results

### 3.1. Patient Characteristics

Our retrospective analysis included 134 participants, comprising 72 patients with AP and 62 control subjects. Of the 72 patients with AP (37 males, 35 females, mean age 65 ± 16 years), 49 had interstitial edematous and 23 necrotizing AP (68% vs. 32%). The characteristics of patients with AP are listed in Table 1.

Regarding age and gender, there was no significant difference between the AP groups and the control group. Within the AP group, patients with interstitial edematous AP were significantly older than patients with necrotizing AP (68 ± 13 years vs. 59 ± 19 years; *p* = 0.036). There was no significant difference between genders regarding the type of AP. The interstitial edematous AP was present in 24 male and 25 female patients, and the necrotizing AP in 13 male and 10 female patients (*p* > 0.05).

The mean BMI (kg/m^2^) was higher than 25 kg/m^2^ in all three groups. The BMI was significantly higher in male patients with AP in both the interstitial edematous and the necrotizing groups when compared to the control group (29.4 ± 4.3 kg/m^2^ and 30.0 ± 4.6 kg/m^2^ vs. 26.2 ± 4.1 kg/m^2^; *p* < 0.05). However, there was no significant difference in BMI between the two AP groups in male patients (*p* > 0.05). The BMI between the female patients among all three groups did not significantly differ (*p* > 0.05).

### 3.2. DECT Scan Analysis

Interobserver agreement was excellent for the assessment of necrosis in patients with AP, and ROI measurements of attenuation values (VNC and mixed HU), ID, and FF in all participants, ranging from 0.982 to 0.995 (all *p* < 0.001).

**Figure 3:**
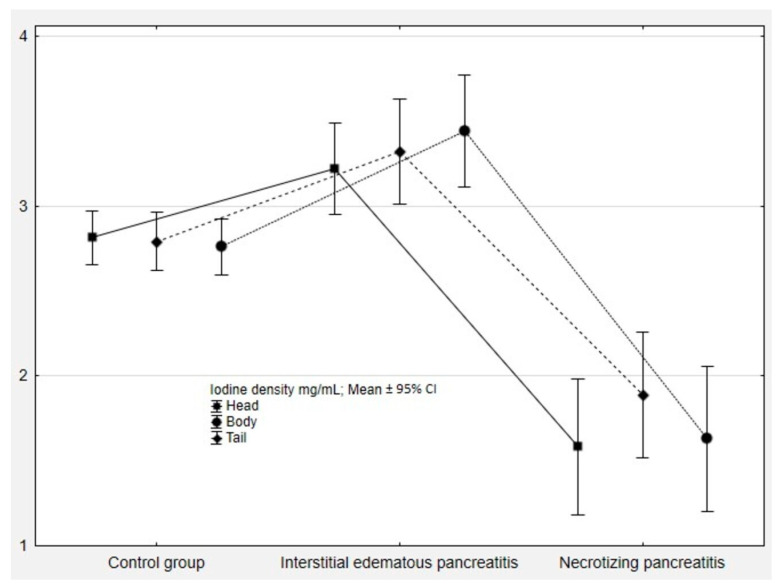
Iodine density values in the control group and patients with acute pancreatitis. CI, confidence interval.

The quantitative DECT measurements in the control group, acute interstitial edematous, and necrotizing pancreatitis groups are shown in Table 2. Iodine density was significantly higher in patients with interstitial edematous AP in comparison to patients with necrotizing AP and control subjects (both *p* < 0.05), as shown in Figure 3.

Necrosis was significantly more frequent in severe AP compared to the mild and moderately severe pancreatitis groups in the body (*p* = 0.011) and tail segments (*p* < 0.001), while the incidence of necrosis did not differ significantly between groups in the head segment (*p* = 0.365).

**Figure 4:**
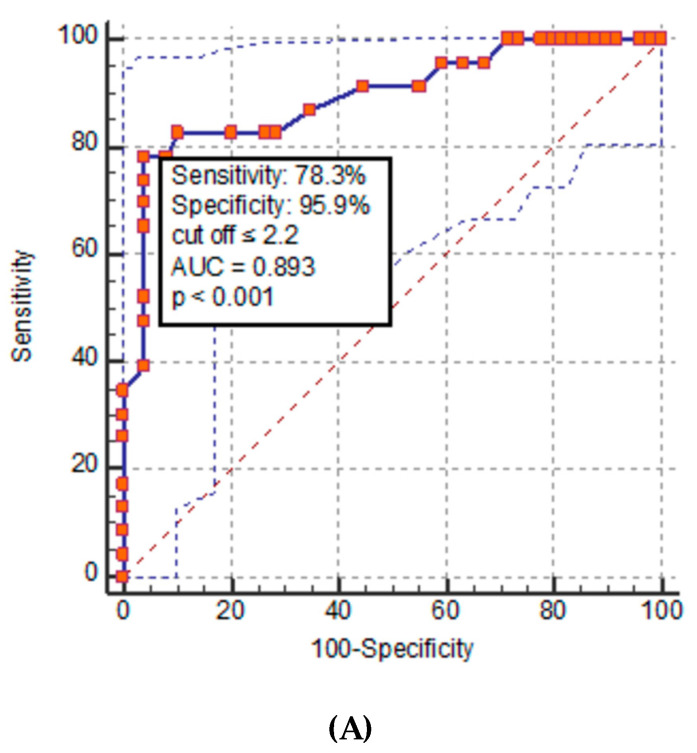

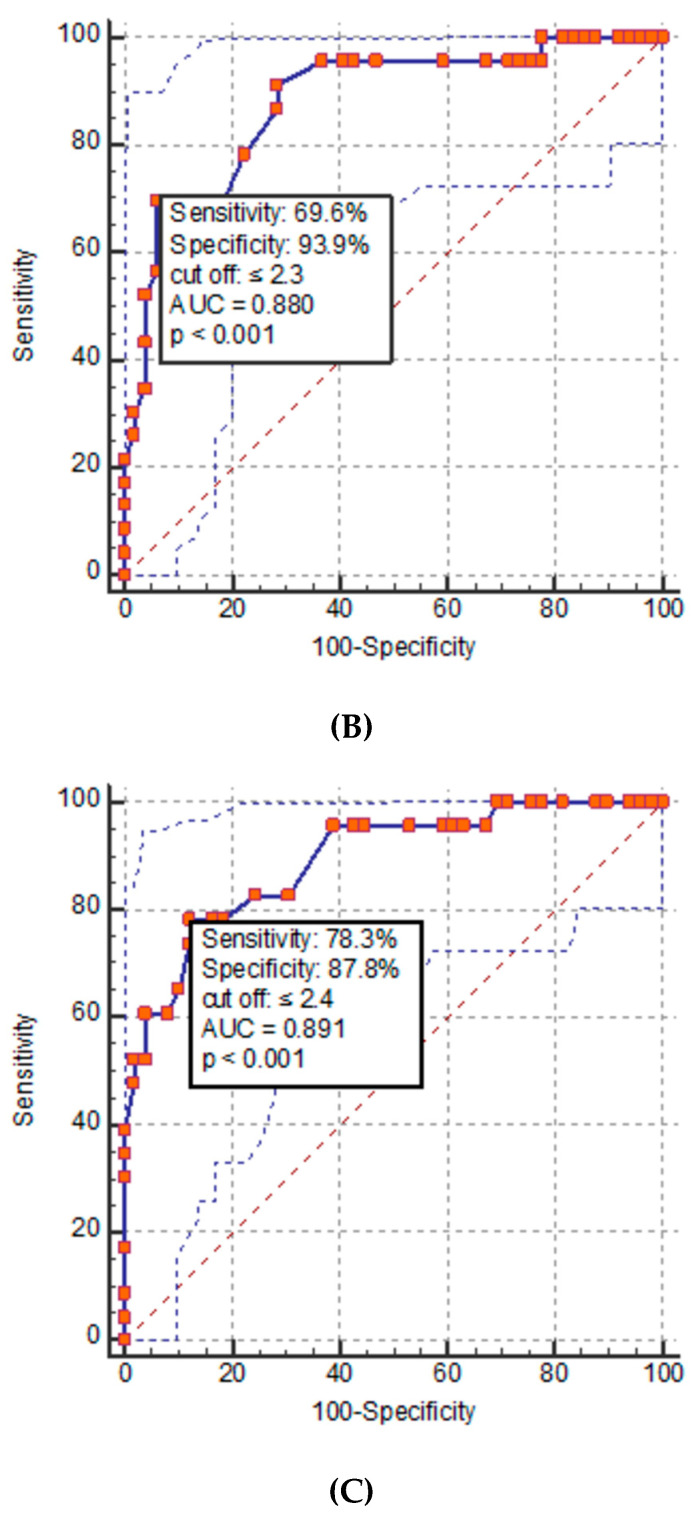
Diagnostic accuracy and discriminative capacity of iodine density value to differentiate interstitial edematous from necrotizing pancreatitis in head (**A**), body (**B**), and tail (**C**) segments of the pancreas. AUC, area under the curve.

If only non-necrotic segments were considered, the difference to the control group was only significantly lower in the pancreatic head (2.3 ± 0.7 mg/mL vs. 2.8 ± 0.6 mg/mL; *p* < 0.05). Non-necrotic segments in the body and tail of the pancreas had a non-significant lower ID compared to the control group. The ROC curve analysis revealed that the thresholds of ID values for detecting pancreatic tissue necrosis were ≤2.2, ≤2.3, and ≤2.4 mg/mL (AUC between 0.880 and 0.893, *p* > 0.05) for the head, body, and tail, respectively (Figure 4).

The FF was significantly higher in the head and body segments in the interstitial edematous AP and necrotizing AP groups compared to the control group (Table 2). In the tail segment, the difference was only significant in the necrotizing AP group (*p* = 0.028).

There was no significant difference in mixed HU attenuation values between the interstitial edematous AP and the control group in all three segments (Table 2).

The VNC values in the head and body segments of the pancreas were significantly lower in both the interstitial edematous and necrotizing groups compared to the control group in the head segment (26.2 ± 18.7 HU and 25.6 ± 12.4 HU vs. 33.0 ± 14.0 HU; *p* = 0.039) and in the body segment (20.1 ± 15.8 HU and 21.1 ± 16.0 HU vs. 30.6 ± 16.9 HU; *p* = 0.002), without significant difference between pancreatitis groups. In the tail segment, values were significantly lower only in the necrotizing group compared to the control group (18.6 ± 15.3 vs. 32.6 ± 12.5; *p* < 0.001).

There was a significant correlation between mixed HU and FF, ranging from −0.565 to −0.638 (all *p* < 0.001) in the head, body, and tail segments, and FF and ID values did not correlate significantly regardless of the segment (all *p* > 0.05).

### 3.3. Clinical and Radiological Evaluation of Disease Severity

According to the APACHE II score, half of the patients with AP had a score below eight and half above eight (36 vs. 36). The APACHE II score was almost equal for both groups, the interstitial edematous AP group and the necrotizing AP group (9.5 vs. 9.3). The mCTSI score was significantly higher in the necrotizing group than in the interstitial edematous group (8.2 ± 1.9 vs. 3.5 ± 1.9; *p* < 0.001). APACHE II and mCTSI showed a significant correlation for the necrotizing group (*p* = 0.011), but there was no correlation for the interstitial edematous AP group (*p* = 0.328). There was a significant negative correlation between the mCTSI score and ID in the body segment for both AP groups (*p* < 0.05). The negative correlation was also significant in the head segment when it came to the interstitial edematous group, and in the tail segment when it came to the necrotizing group (both *p* < 0.05).

## 4. Discussion

Initial CECT is not usually necessary for the diagnosis of AP. It should be performed in patients with clinically severe AP and when developing complications are suspected [10]. Approximately 5–10% of patients with AP develop necrosis [3]. The higher proportion of the necrotizing type of AP in our study group (32%) might result from the fact that not all patients with AP underwent DECT (patients with a mild clinical course or patients who were unable to undergo CT because of their condition).

We decided to perform a DECT scan in the pancreatic phase 40 s after the intravenous administration of contrast media, as in the pancreatic phase, the enhancement of pancreatic parenchyma is the most intensive [4,24]. Although some authors suggest scanning patients with AP in a portal-venous phase [25], others propose scanning in a pancreatic phase [26]. In the DECT study conducted by Hu et al. [27], the sensitivity and specificity of ID values were better in an arterial than in a portal-venous phase. Nevertheless, results from DECT studies conducted by Martin et al. [19] in a pancreatic phase and by Mahmoudi et al. [20] in a portal-venous phase were similar regarding attenuation, ID, and FF values.

According to our results, a higher BMI was a predisposing factor for the development of AP in male patients, both interstitial edematous and necrotizing types. Mao et al. [28] investigated thirty potential modifiable risk factors associated with AP and recognized BMI as one of them. Even though BMI has been a risk factor for worsening AP [29], a higher BMI alone cannot predict the severity of AP [30], which is consistent with our results (no difference between the interstitial edematous and the necrotizing group).

Our results have shown that attenuation values (mixed HU) are not sensitive enough to detect interstitial edematous AP and do not differ from those of the control group. The results are in accordance with Martin et al. [19], who reported no significant differences in attenuation values between normal and inflammatory pancreatic parenchyma.

On the other hand, in our study, ID significantly differentiates the interstitial edematous AP group from the control group. The vascular theory of the development and progression of AP involves impaired pancreatic microcirculation together with altered tissue perfusion and the activation of inflammatory processes, and has a role in the complex pathogenesis of AP [31]. The increase in blood flow with homogeneous capillary perfusion is responsible for developing hyperemia in interstitial edematous AP. In contrast, a progressive decrease in perfused capillaries was found in necrotizing AP [32,33]. Kinnala et al. [34] also showed that in a mild AP, pancreatic oxygenation increases, while a severe form has markedly impaired tissue perfusion. Our results confirmed the absence of hyperemia in the necrotizing AP group, even in the non-necrotic parenchymal segments, in contrast to the interstitial edematous AP group. In fact, the non-necrotic head segment demonstrated significant hypoperfusion in patients with necrotizing AP compared to the control group. In our study, the ROC curve analysis suggested the thresholds of ID of ≤2.2, ≤2.3, and ≤2.4 mg/mL in the pancreatic head, body, and tail, respectively, to be reliable thresholds for discrimination between interstitial edematous and necrotizing AP. Previous studies demonstrated lower ID, with a threshold of ≤2.1 mg/mL in all patients with AP, when compared to normal pancreatic parenchyma [19], and in severe AP, ID was lower than ≤1.63 mg/mL [20]. The severity assessment of AP [9] includes extrapancreatic complication evaluation, as well as pancreatic tissue inflammation and possible necrosis evaluation, which could explain the difference.

Dual-energy CT is a well-established imaging technique for quantifying FF in the pancreas [35]. Pancreatic dysfunction is related to the accumulation of fat in pancreatic tissue. Fat distribution through pancreatic parenchyma is not homogeneous, and its deposition might be related to older age, diabetes, obesity, and diseases such as cystic fibrosis and Shwachman–Diamond syndrome [36]. Patients with type 2 diabetes mellitus (T2DM) have a higher degree of FF in all parts of the pancreas (head, body, and tail) when compared to the control group [37]. FF varies between the head, body, and tail of the pancreas, where an increased FF in the tail of the pancreas may be related to the risk of developing T2DM [38]. Our study data suggest that the FF of the pancreatic tissue was significantly higher in the head and body segments in all patients with AP. In addition, FF was significantly higher in the tail segment only in patients with necrotizing AP. Previous DECT studies of AP found no significant differences in FF between pancreatitis groups, and between patients with AP and the control group [19,20]. In our study, the fat content in the head, body, and tail of the pancreas was measured separately, which may explain the difference in FF between the pancreatitis groups and the control group. The inability of mixed HU to differentiate interstitial edematous pancreatitis from normal pancreatic parenchyma might be the consequence of the impact of higher FF on attenuation values. On the contrary, ID remained unaffected by tissue FF.

Half of all patients with AP referred to DECT had clinically severe disease. The APACHE II score is based on clinical and laboratory findings, and a score of eight or higher indicates severe AP [1,6]. The mCTSI score focuses on the CT features of pancreatitis, the possible necrosis of pancreatic tissue, and extrapancreatic complications [9]. We demonstrated a negative correlation between mCTSI and ID not only in the necrotizing AP group (body and tail segments) but also in the interstitial edematous group in the pancreatic head and body segments, providing a link between morphologic CT features and pancreatic tissue perfusion. APACHE II showed a significant correlation with the mCTSI score only in the necrotizing AP group. Compared to APACHE II, mCTSI more accurately diagnoses clinically severe AP [10,39].

Contrast-enhanced CT and MRI could be used in accessing pancreatic tissue necrosis [10]. An MRI, as an alternative to CECT, allows an even better evaluation of bile and pancreatic ducts and hemorrhage [40]. However, in critical patients, CECT is the preferred imaging modality due to accessibility, shorter examination time, and no need for cooperation, with DECT allowing for a better assessment of pancreatic necrosis than conventional CECT.

There are some apparent limitations to our research. First, it was a retrospective, single-center study. Second, the readers knew about the existence of AP, or lack thereof, which may lead to potential bias. Also, we did not analyze the interobserver and intraobserver reliability agreement for correlating the morphologic severity of AP, which may be considered in further research. Finally, not all hospitalized patients with AP undergo CT exams, which might explain the higher proportion of the necrotizing type of AP in this study.

In conclusion, DECT has the potential to accurately quantify FF and ID in the pancreatic tissue. There is an apparent association between FF and attenuation values. On the contrary, an elevated FF did not influence the ID values. We observed significant variations in the ID of pancreatic tissue measured by means of DECT, which showed tissue hyperperfusion in patients with interstitial edematous AP and hypoperfusion in patients with necrotizing AP, and with thresholds of ID that allow for differentiation between morphologic types of AP. The hypoperfusion of the pancreatic head in the necrotizing AP group was significant compared to the control group, even without necrosis in this segment. We also detected an unevenly increased FF of the tissue in different segments of the pancreas in patients with AP. In the tail segment, it was increased only in the necrotizing AP group. BMI was significantly higher in male patients with AP compared to the control group. There was a significant correlation between mCTSI and ID in two out of three different pancreatic segments in both types of AP.

## Figures and Tables

**Table 1:** The characteristics of patients with acute pancreatitis.

Patients with AP	
All (N)	72
Male (N)	37
Female (N)	35
Age (years)	65 ± 16
Interstitial edematous pancreatitis (N)	49
Necrotizing pancreatitis (N)	23
BMI (kg/m^2^)	
Male	29.6 ± 4.3
Female	28.0 ± 6.8
mCTSI	
Mild AP (N)	21
Moderately severe AP(N)	31
Severe AP (N)	20
Length of hospital stay (days)	
Interstitial edematous pancreatitis	9 ± 4
Necrotizing pancreatitis	23 ± 18
In-hospital mortality	
Interstitial edematous pancreatitis (N)	0
Necrotizing pancreatitis (N)	3

AP, acute pancreatitis; BMI, body mass index; mCTSI, modified CT severity index.

**Table 2:** Quantitative DECT measurements in the control, acute interstitial edematous, and necrotizing pancreatitis groups.

	Control Group (N = 62)	Interstitial Edematous Pancreatitis (N = 49)	Necrotizing Pancreatitis (N = 23)	*p*-Value
	Mean ± SD			
Iodine density (mg/mL)				
Head	2.8 ± 0.6	3.2 ± 0.9	1.6 ± 0.9	<0.001 *
Body	2.8 ± 0.7	3.3 ± 1.1	1.9 ± 0.9	<0.001 *
Tail	2.8 ± 0.6	3.4 ± 1.1	1.6 ± 0.9	<0.001 *
FF (%)				
Head	13.1 ± 9.4	20.1 ± 10.2	18.4 ± 8.6	<0.001 *
Body	14.3 ± 11.6	22.3 ± 11.1	23.1 ± 11.3	<0.001 *
Tail	14.1 ± 15.5	16.9 ± 9.4	22.5 ± 10.3	0.028 *
Mixed HU				
Head	89.3 ± 20.8	83.4 ± 23.8	54.8 ± 23.3	<0.001 *
Body	85.7 ± 23.1	82.4 ± 24.5	54.6 ± 23.9	<0.001 *
Tail	88.8 ± 18.9	88.8 ± 18.9	78.0 ± 30.4	<0.001 *
VNC (HU)				
Head	33.0 ± 14.0	26.2 ± 18.7	25.6 ± 12.4	0.039 *
Body	30.6 ± 16.9	20.1 ± 15.8	21.1 ± 16.0	0.002 *
Tail	32.6 ± 12.5	28.0 ± 10.8	18.6 ± 15.3	<0.001 *

* Indicates statistically significant results. DECT, dual-energy computed tomography; FF, fat fraction; HU, Hounsfield units; VNC, virtual-non-contrast.

## Data Availability

All data generated or analyzed during this study are included in this article. Further enquiries can be directed to the corresponding author.
